# Supplementary material for: The Impact of Pre-Chemotherapy Body Composition and Immunonutritional Markers on Chemotherapy Adherence in Stage III Colorectal Cancer Patients
Source: J Clin Med. 2023 Feb 10;12(4):1423. doi: 10.3390/jcm12041423 (PMC9962672; doi:10.3390/jcm12041423)
Supplement: Supplementary file 1 [file jcm-12-01423-s001.zip › jcm-2184843-supplementary.pdf]

Table S1. Causes of dose reduction or chemotherapy discontinuation divided according to toxicity grade in low RDI group

	Toxicity grades				Total patients (No)
**Cause**	**Grade 1 (No)**	**Grade 2 (No)**	**Grade 3 (No)**	**Grade 4 (No)**	
Peripheral sensory neuropathy	0	4	4	0	8
Decreased Neutrophil count	0	0	3	3	6
Nausea	0	4	0	0	4
Fatigue	0	1	1	0	2
Lung infection	0	0	0	1	1
Abdominal pain	0	0	1	0	1
**Total counts (No)**	0	9	8	4	22

Table S2. Causes of dose reduction or chemotherapy discontinuation divided according to toxicity grade in high RDI group

	Toxicity grades				Total patients (No)
**Cause**	**Grade 1 (No)**	**Grade 2 (No)**	**Grade 3 (No)**	**Grade 4 (No)**	
Peripheral sensory neuropathy	0	6	0	0	6
Peripheral motor neuropathy	0	1	0	0	1
Neutrophil count decreased	0	0	5	0	5
Nausea	4	3	0	0	7
Fatigue	0	2	0	0	2
Myalgia	0	1	0	0	1
Abdominal pain	0	1	0	0	1
Urticaria	1	2	0	0	3
Dyspnea	0	0	1	0	1
Generalized edema	1	1	0	0	2
Diarrhea	0	1	0	0	1
Syncope	0	0	1	0	1
Intestinal stoma site bleeding	0	0	1	0	1
**Total counts (No)**	6	18	8	0	32

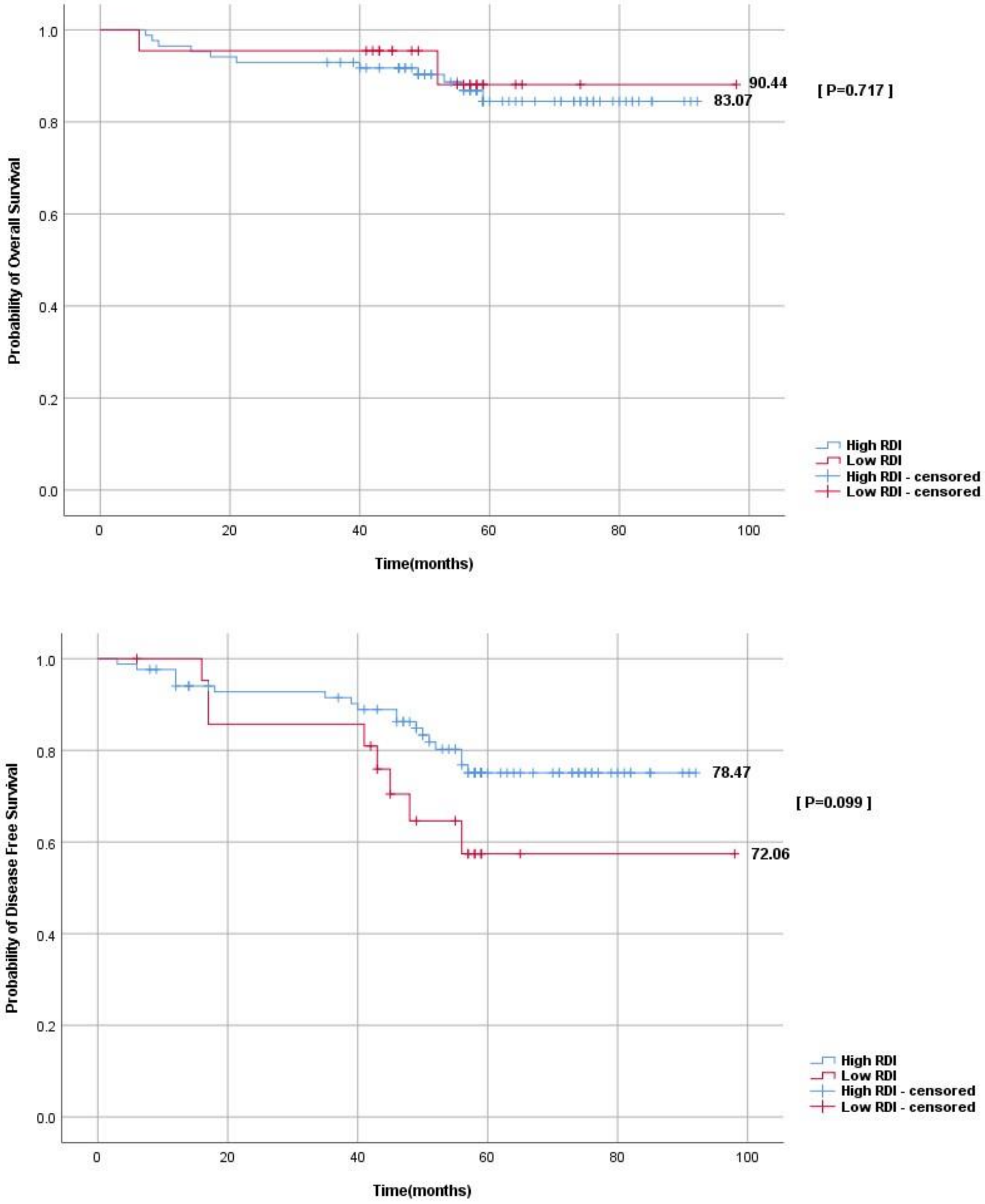

Figure S1. Five-year overall survival and disease free survival according to RDI
